# OSdlbcl: An online consensus survival analysis web server based on gene expression profiles of diffuse large B‐cell lymphoma

**DOI:** 10.1002/cam4.2829

**Published:** 2020-01-09

**Authors:** Huan Dong, Qiang Wang, Guosen Zhang, Ning Li, Mengsi Yang, Yang An, Longxiang Xie, Huimin Li, Lu Zhang, Wan Zhu, Shuchun Zhao, Haiyu Zhang, Xiangqian Guo

**Affiliations:** ^1^ Department of Predictive Medicine, Institute of Biomedical Informatics Cell Signal Transduction Laboratory Bioinformatics Center Henan Provincial Engineering Center for Tumor Molecular Medicine, School of Software School of Basic Medical Sciences Henan University Kaifeng China; ^2^ Department of Anesthesia Stanford University School of Medicine Stanford CA USA; ^3^ Department of Pathology Stanford University School of Medicine Stanford CA USA

**Keywords:** diffuse large B‐cell lymphoma, OSdlbcl, prognostic biomarker, survival analysis

## Abstract

Diffuse large B‐cell lymphoma (DLBCL) is the most common subtype of non‐Hodgkin lymphoma (NHL) and is a clinical, pathological, and molecular heterogeneous disease with highly variable clinical outcomes. Currently, valid prognostic biomarkers in DLBCL are still lacking. To optimize targeted therapy and improve the prognosis of DLBCL, the performance of proposed biomarkers needs to be evaluated in multiple cohorts, and new biomarkers need to be investigated in large datasets. Here, we developed a consensus **O**nline **S**urvival analysis web server for **D**iffuse **L**arge **B**‐**C**ell **L**ymphoma, abbreviated **OSdlbcl**, to assess the prognostic value of individual gene. To build OSdlbcl, we collected 1100 samples with gene expression profiles and clinical follow‐up information from The Cancer Genome Atlas (TCGA) and Gene Expression Omnibus (GEO) databases. In addition, DNA mutation data were also collected from the TCGA database. Overall survival (OS), progression‐free survival (PFS), disease‐specific survival (DSS), disease‐free interval (DFI), and progression‐free interval (PFI) are important endpoints to reflect the survival rate in OSdlbcl. Moreover, clinical features were integrated into OSdlbcl to allow data stratifications according to the user's special needs. By inputting an official gene symbol and selecting desired criteria, the survival analysis results can be graphically presented by the Kaplan‐Meier (KM) plot with hazard ratio (HR) and log‐rank *p* value. As a proof‐of‐concept demonstration, the prognostic value of 23 previously reported survival associated biomarkers, such as transcription factors *FOXP1* and *BCL2*, was evaluated in OSdlbcl and found to be significantly associated with survival as reported (HR = 1.73, *P* < .01; HR = 1.47, *P* = .03, respectively). In conclusion, OSdlbcl is a new web server that integrates public gene expression, gene mutation data, and clinical follow‐up information to provide prognosis evaluations for biomarker development for DLBCL. The OSdlbcl web server is available at https://bioinfo.henu.edu.cn/DLBCL/DLBCLList.jsp.

## Graphical abstract

To assess and identify appropriate prognostic biomarkers for diffuse large B‐cell lymphoma, we integrated public gene expression data and clinical follow‐up information and developed the consensus online survival analysis web server OSdlbcl for researchers and clinicians.

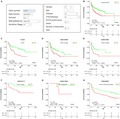

## 1 INTRODUCTION

Diffuse large B‐cell lymphoma (DLBCL) is the most common subtype of non‐Hodgkin lymphoma (NHL), accounting for 30%‐40% of the NHL.^1^, ^2^ DLBCL is a clinical, pathological, and molecular heterogeneous disease, patients of which have highly variable clinical outcomes.^3^ The current complex classification of DLBCL is presented in World Health Organization (WHO).^4^, ^5^ Although this disease is curable, 20%‐30% of DLBCL patients still experience relapse or refractory disease.^6^, ^7^ To assist clinical treatment, prognostic biomarkers are being investigated to optimize targeted therapy and to predict the prognosis of high‐risk DLBCL patients.^8^

So far, some unfavorable prognostic factors for DLBCL have been reported in previous studies, such as high international prognostic index (IPI), MYC rearrangement, double‐hit lymphoma, double‐expression lymphoma, and high p53 and CD5 expression.^1^, ^6^ However, more reliable biomarkers with high repeatability and predictive power to diagnose high‐risk patients are needed to facilitate the development of alternative treatment strategies for DLBCL.^9^ Using microarray or RNA‐Seq technologies, the discovery of prognostic biomarkers at the transcriptional level is one main achievement of cancer genomics.^10^ Despite the availability of numerous expression data and the corresponding clinical information in the public database to date, a web server or tool that could quickly evaluate the prognostic value of potential DLBCL biomarkers is still lacking.

In this study, we collected the gene expression profiles and clinical information of 1100 DLBCL patients from seven independent cohorts from the TCGA and GEO databases. We developed an online consensus survival analysis web server, named OSdlbcl, to assess the prognostic value of interested genes. This web server will facilitate the development and validation of new prognostic biomarkers in DLBCL.

## 2 METHODS AND MATERIALS

### 2.1 Dataset collection

RNA expression profiling data and clinical follow‐up information of DLBCL patients were downloaded from two major sources, including TCGA (https://portal.gdc.cancer.gov/) and GEO (http://www.ncbi.nlm.nih.gov/geo/). For TCGA data, Level 3 RNASeq data (HiSeqV2) with clinical information of DLBCL patients were downloaded. To gather the data in GEO, the searching keywords of “diffuse large B‐cell lymphoma” or “DLBCL” and “survival” were used in GEO database. Only datasets that contain ≥ 25 cases with available gene expression profiles and clinical survival information were selected. In addition, DNA mutation data of DLBCL patients with clinical follow‐up information were downloaded from TCGA.

### 2.2 System implementation and server setup

OSdlbcl was developed as previously described.^11^, ^12^, ^13^, ^14^ The Kaplan‐Meier (KM) plot of cumulative survival probability over time is a hallmark of biomedical survival analysis.^11^ The log‐rank test is popularly used to compare survival experience between groups. Thus, the KM plot and log‐rank test were used to estimate the risk of the events in OSdlbcl. In short, J2EE (Java 2 Platform Enterprise Edition) architecture and MySQL server were used for integrating gene expression, DNA mutation, and clinical data. The dynamic web interfaces were written in HTML 5.0 and hosted by Tomcat in a Windows server. As the web server is “out‐of‐the‐box,” when users input an official gene symbol, the statistical analyses will be performed by the R package “survival” to produce the KM curves with hazard ratio (HR, 95% confidence interval) and log‐rank *p* value. OSdlbcl is available at https://bioinfo.henu.edu.cn/DLBCL/DLBCList.jsp.

### 2.3 Evaluation of previously reported prognostic biomarkers

To evaluate the prognostic power of previously reported prognostic biomarkers, keywords including “Diffuse large B‐cell lymphoma” or “DLBCL,” “gene expression,” and “survival” or “prognosis” were used in the PubMed search engine. In total, 23 biomarkers were collected, and the prognostic values of these reported DLBCL biomarkers were analyzed by OSdlbcl.

## 3 RESULTS

### 3.1 Clinical characteristics of DLBCL datasets used in OSdlbcl

To establish OSdlbcl web server, we downloaded one TCGA cohort and six GEO cohorts with gene expression profiles and clinical follow‐up information (Table 1).^15^, ^16^, ^17^, ^18^, ^19^, ^20^, ^21^, ^22^, ^23^ A total of 1100 unique DLBCL cases were collected, all of which have available gene expression profiling data and clinical follow‐up survival information. Overall survival (OS) is the most important endpoint for the clinical outcomes in OSdlbcl.^11^ Moreover, we also collected survival terms including progression‐free survival (PFS), disease‐specific survival (DSS), disease‐free interval (DFI), and progression‐free interval (PFI) for the “survival” option in OSdlbcl web server. Before survival analysis, users could choose the relevant clinical characterizations, such as Ann Arbor stage, age, ECOG performance status, gender, IPI group, or number of extranodal sites, to narrow the analysis in a subgroup of DLBCL patients. The main clinical characteristics of these cohorts in OSdlbcl are shown in Table 1. DLBCL samples in OSdlbcl were collected from various areas, but most of them were from North America and Europe. Most of the DLBCL patients are male and at an elder age with the median age over sixty. All of the 1100 patients have OS data with a median OS of 28.50 months, while 267 patients from TCGA and 29 patients from http://www.ncbi.nlm.nih.gov/geo/query/acc.cgi?acc=GSE21864 also have PFS data. In addition, the 267 patients from TCGA have DSS, DFI, and PFI data as well. The total number of death events is 437, which is 39.72% of the total patients in OSdlbcl (Table 1).

**Table 1:** Clinical characteristics of seven independent DLBCL datasets used in OSdlbcl

Data source	Platform	Sample size	No. of deaths	Median age	Median OS (months)	Gender (% male)	CHOP/R‐CHOP	Stage (% I/II/III/IV/NA)	Area	Survival terms	References
TCGA	HiSeqV2	267	101	62	60.70	54.89	—	16.67/29.17/26.25/25.83/1.67	Asian and America	OS, PFS, DFI, PFI, DSS	[11]
http://www.ncbi.nlm.nih.gov/geo/query/acc.cgi?acc=GSE10846	GPL570	414	165	63	28.50	58.45	181/233	15.94/29.47/23.43/29.23/1.93	North America and Europe	OS	[15, 16]
http://www.ncbi.nlm.nih.gov/geo/query/acc.cgi?acc=GSE53786	GPL570	119	44	63	23.04	57.14	45/71	14.29/29.41/26.05/27.73/2.52	North America	OS	[17]
http://www.ncbi.nlm.nih.gov/geo/query/acc.cgi?acc=GSE57611	GPL96	31	18	66	10.90	45.16	21/‐	9.68/25.81/38.71/6.45/19.35	Germany	OS	[18]
http://www.ncbi.nlm.nih.gov/geo/query/acc.cgi?acc=GSE32918	GPL8432	172	88	—	52.45	62.87	—	—	North America	OS	[19, 20]
http://www.ncbi.nlm.nih.gov/geo/query/acc.cgi?acc=GSE21846	GPL1078	29	4	—	18.00	—	—	—	Spain, Italy, USA	OS, PFS	[21]
http://www.ncbi.nlm.nih.gov/geo/query/acc.cgi?acc=GSE34171	GPL570	68	17	—	47.56	—	—	—	North America	OS	[22, 23]
Total		1100	437		28.50						

### 3.2 Application of OSdlbcl web server

**Figure 1:**
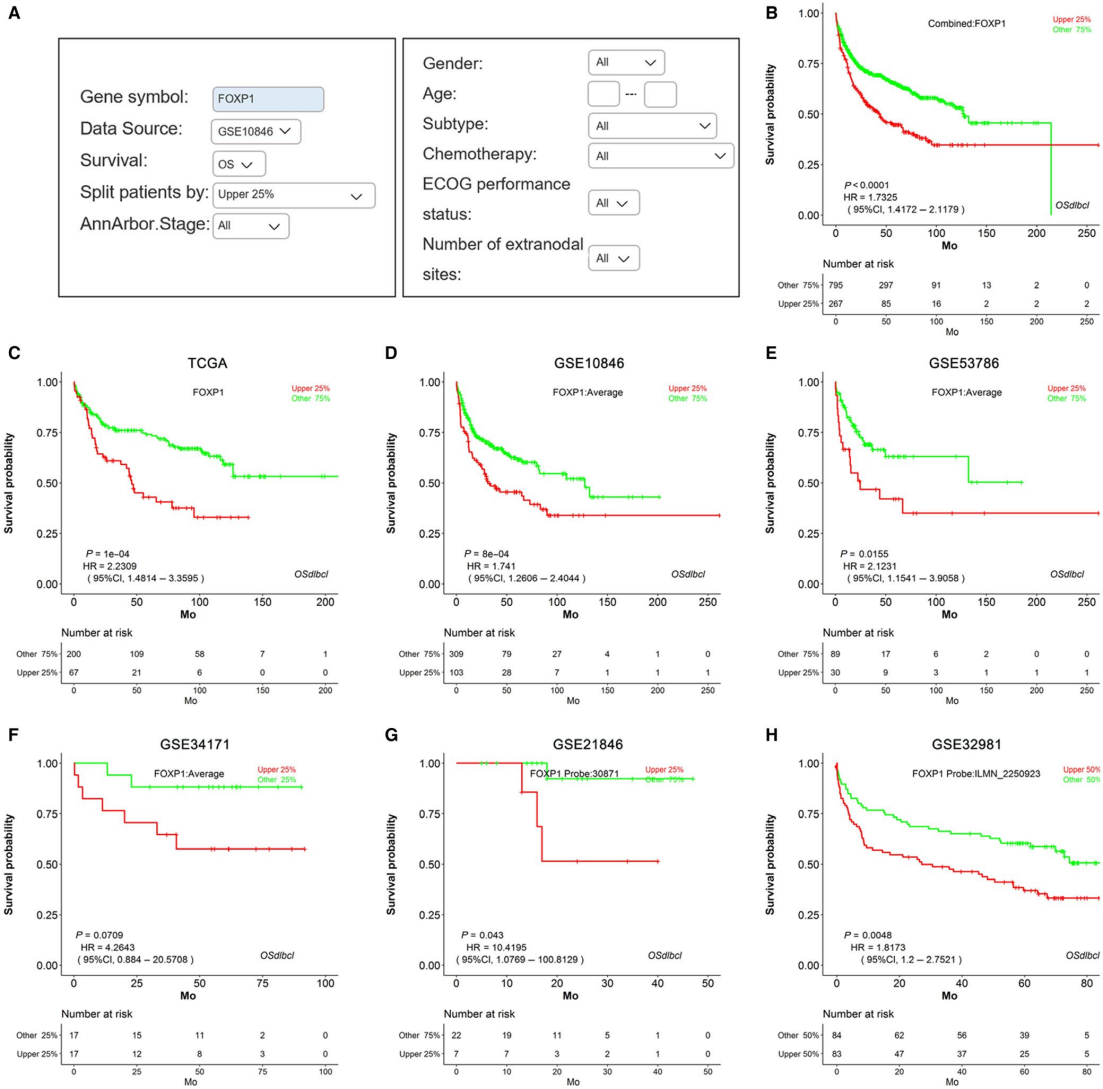
Kaplan‐Meier survival curves according to the gene expression of *FOXP1* in OSdlbcl. A, The options of prognostic analysis using *FOXP1* as an example in OSdlbcl. B, The output web page for *FOXP1* prognosis analysis using the combined cohorts with all patients’ data in OSdlbcl. C‐H, The OSdlbcl output of gene *FOXP1* in a single cohort

To evaluate the prognostic value of genes in OSdlbcl web server, users first input a gene symbol, choose either individual cohort or combined cohorts, select one of the survival outcome types (OS, DSS, DFI, PFI, or PFS), and designate a gene expression cutoff value that will be used to split the DLBCL patients for KM analysis 24 (Figure 1A). Users could also limit survival analysis to focus on a subgroup of DLBCL patients by setting Ann Arbor stage, age, ECOG performance status, gender, IPI group, or number of extranodal sites (Figure 1A).^11^, ^25^ Finally, users could click the “Kaplan‐Meier plot” button to run KM analysis. And then, the OS, DSS, DFI, PFI, or PFS of the gene in query is determined and graphically displayed with HR (95% CI) and log‐rank *p* on output web page (Figure 1).

### 3.3 Evaluation of previously reported DLBCL prognostic biomarkers in OSdlbcl

Independent validation across different cohorts is of great importance for biomarker development, and OSdlbcl is a web server that could facilitate the cross‐validation of the prognostic value of biomarkers across seven DLBCL cohorts. To determine the prognosis performance of OSdlbcl, we collected 23 previously published DLBCL prognostic factors at the mRNA or protein level and tested their prognostic power in OSdlbcl at the mRNA level (Table 2, Figure 1).^2^, ^9^, ^26^, ^27^, ^28^, ^29^, ^30^, ^31^, ^32^, ^33^, ^34^, ^35^, ^36^, ^37^, ^38^, ^39^, ^40^, ^41^, ^42^, ^43^, ^44^, ^45^ The forest plots more effectively presented the comparison results from references and OSdlbcl (Figure S1). Results showed that about 52% (12 out of 23) of biomarkers have significant prognostic values in the combined cohorts, while the remaining biomarkers (11 out of 23) present significant prognostic values in one or more cohorts (Table 2). Among these biomarkers, *FOXP1* and *BCL2* have been shown to associate with worse prognosis in DLBCL cases.^9^, ^42^, ^43^, ^46^ In OSdlbcl, we found the *FOXP1* (HR = 1.73, *P* < .01) and BCL2 (HR = 1.47, *P* = .03) (Table 2, Figure 1) were consistent to be adverse prognostic biomarkers for DLBCL patients.

**Table 2:** Validation of previously reported prognostic biomarkers in OSdlbcl

Gene symbol	OS in OSdlbcl				OS in reference				
	HR (95% CI)	*P* value	Cutoff	Data source	HR (95% CI)	*P* value	Sample (n)	Level	References
PATZ1	3.02 (1.12‐8.13)	.03	Upper 25% VS Lower 75%	http://www.ncbi.nlm.nih.gov/geo/query/acc.cgi?acc=GSE57611	—	.02	470	mRNA	[26]
MYC	2.06 (1.43‐2.97)	<.01	Upper 25% VS Lower 25%	Combined	—	.01	120	Protein	[27]
CFLAR	1.86 (1.10‐3.14)	.02	Upper 25% VS Lower 25%	TCGA	—	.03	71	Protein	[28]
FOXP1	1.73 (1.41‐2.11)	<.01	Upper 25% VS Lower 75%	Combined	—	.02	35	Protein	[9]
FBXW7	1.72 (1.00‐2.97)	<.05	Upper 30% VS Lower 30%	http://www.ncbi.nlm.nih.gov/geo/query/acc.cgi?acc=GSE32918	—	<.01	164	mRNA	[29]
RGS1	1.70 (1.10‐2.63)	.02	Upper 25% VS Lower 75%	http://www.ncbi.nlm.nih.gov/geo/query/acc.cgi?acc=GSE32918	—	.04	240	Protein	[30]
S1PR1	1.63 (1.02‐2.60)	.04	Upper 30% VS Lower 30%	Combined	1.12 (1.04‐1.20)	.03	68	Protein	[31]
SPN	1.54 (1.01‐2.35）	.04	Lower 25% VS Upper 75%	TCGA	3.64 (2.18‐6.08)	<.01	160	mRNA	[32]
BCL2	1.47 (1.04‐2.07)	.03	Upper 30% VS Lower 30%	Combined	3.13 (1.23‐7.97)	.02	79	Protein	[33]
CXCR4	1.34 (1.02‐1.75)	.03	Upper 50% VS Lower 50%	Combined	1.79 (1.06‐3.02)	.03	233	mRNA	[34]
eIF2B5	1.31 (1.03‐1.66)	.03	Upper 30% VS Lower 30%	Combined	—	<.01	58	mRNA	[35]
SLIT2	0.70 (0.49‐0.97)	.03	Upper 30% VS Lower 30%	Combined	—	.04	107	Protein	[36]
CXCL9	1.23 (0.85‐1.79)	.27	Upper 25% VS Lower 25%	TCGA	—	<.05	95	Protein	[39]
TP53	0.69 (0.49‐0.97)	.03	Upper 25% VS Lower 25%	Combined	—	<.01	—	Protein	[2]
SORL1	0.68 (0.46‐0.10)	<.05	Upper 25% VS Lower 25%	Combined	—	<.01	133	Serum	[37]
NOTCH3	0.6 (0.45‐0.97)	.04	Upper 25% VS Lower 25%	Combined	—	.07	26	mRNA	[38]
TNFRSF8	0.58 (0.40‐0.84)	<.01	Upper 25% VS Lower 25%	Combined	0.25 (0.08‐0.75)	.01	32	Protein	[40]
IRF8	0.56 (0.34‐0.91)	.02	Upper 30% VS Lower 70%	http://www.ncbi.nlm.nih.gov/geo/query/acc.cgi?acc=GSE32918	—	.02	67	Protein	[41]
CD5	0.56 (0.33‐0.98)	.04	Upper 30% VS Lower 30%	http://www.ncbi.nlm.nih.gov/geo/query/acc.cgi?acc=GSE32918		.02	123	Protein	[42]
MUM1	0.563 (0.33‐0.96)	.03	Upper 30% VS Lower 30%	TCGA	—	.01	71	Protein	[28]
MME	0.55 (0.32‐0.96)	.03	Upper 25% VS Lower 25%	TCGA	—	.03	71	Protein	[28]
S1PR2	0.48 (0.33‐0.70)	<.01	Upper 25% VS Lower 25%	Combined	—	<.01	233	mRNA	[43]
DNMT1	0.35 (0.19‐0.65)	<.01	Upper 25% VS Lower 75%	TCGA	2.40 (2.38‐10.02)*	<.01	230	Protein	[44]

* Lower gene expression compared with higher gene expression in the literature data.

### 3.4 Determination of the prognostic value of DNA mutation in OSdlbcl

**Figure 2:**
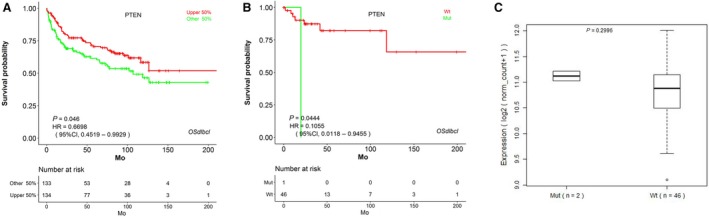
Kaplan‐Meier survival estimates according to the gene expression and mutation status of *PTEN* in OSdlbcl. A‐B, The output web page for *PTEN* prognosis analysis of RNA expression (A) and DNA mutation (B) using the TCGA cohort in OSdlbcl. C, The output web page for analysis *PTEN* expression according to mutation status in OSdlbcl

In addition to gene expression, gene sequence variation is another common type of prognostic factor.^47^ In order to implement the prognosis analysis based on gene sequence variation, we have collected the DLBCL DNA mutation data from TCGA and implemented them into OSdlbcl web server, by which users could perform the prognosis analysis based on DNA mutation for the input gene. For example, *PTEN* is a tumor suppressor and mutated in a large number of cancers at high frequency, and *PTEN* deletion, mutation, and loss of *PTEN* expression were of clinical significance in de novo DLBCL.^48^ As a result, we investigated the prognostic performance of *PTEN* in OSdlbcl at both RNA and DNA levels, and the results showed that *PTEN* is an independent favorable prognostic factor for OS at the RNA level (HR = 0.67, *P* < .05) (Figure 2A), while *PTEN* mutation is the independent prognostic factor for poorer survival in DLBCL (HR = 0.11, *P* = .04) (Figure 2B). In addition, the gene expression variation between DLBCL cases with wild‐type (Wt) and mutation (Mut) gene types can also be investigated in OSdlbcl (Figure 2C).

## 4 DISCUSSION

DLBCL is a heterogeneous disease with highly variable clinical outcomes.^3^ Efficient biomarkers can help predicting clinical outcomes and identifying high‐risk patients. However, the biomarkers currently used can only reflect a small spectrum of DLBCL patients. Therefore, we developed a user‐friendly online survival analysis web server for researchers and clinicians to assess and identify prognostic biomarkers in DLBCL in a big dataset.

Compared to previously published prognostic biomarker tools such as OncoLnc,^49^ KM plotter,^50^, ^51^, ^52^ and UALCAN,^53^ OSdlbcl has the following advantages. First, OSdlbcl is the first survival analysis web server specifically for DLBCL and contains largest DLBCL sample size (1100 samples) compared to the other databases. Second, the interface of OSdlbcl is very straightforward and easy for the users with no specific bioinformatics training to operate. Also, the survival analysis results can be graphically presented by the KM plot with HR and log‐rank* p* value, which could be used to assess the prognostic value of gene of interest. Third, except for prognosis evaluation at the RNA level, OSdlbcl could also determine the prognosis value of DNA mutation for DLBCL patients. Fourth, OSdlbcl has incorporated the clinical covariates for DLBCL patients including Ann Arbor stage, gender, ECOG performance status, number of extranodal sites, and IPI. Last but not least, 23 previously reported prognostic biomarkers were confirmed in the OSdlbcl web server, which indicated the effectiveness of OSdlbcl, and these previously reported biomarkers may have the potential to be translated into clinical applications. The limitation of OSdlbcl is that the number of DLBCL cases used for DNA mutation survival analysis is too small. However, continuously updating the OSdlbcl database by adding latest gene variation profiles and expression profiles with accurate follow‐up information will help to strengthen the performance of OSdlbcl.

In conclusion, OSdlbcl is a user‐friendly online consensus survival analysis web server to efficiently identify prognostic biomarkers, and the OSdlbcl database will be regularly updated when new DLBCL data are available. Our web servers will well reveal the critical impact of RNA expression and gene variation on the prognosis of DLBCL and are fundamentally important for the future targeted therapy for improving clinical outcomes.

## CONFLICTS OF INTEREST

All authors declare no competing interests.

## AUTHOR CONTRIBUTIONS

XG conceived and directed this project. HD, QW, and GZ collected data and developed the web server. HD, NL, MY, YA, LX, HL, and LZ performed data analysis. WZ, SZ, and HZ contributed to data analysis and paper writing. HD wrote the manuscript with the assistance and approval of all authors.

## Data Availability

This study analyzed the publicly available datasets. These data are available online at http://bioinfo.henu.edu.cn/DLBCL/DLBCLList.jsp.
