# Supplementary material for: OSdlbcl: An online consensus survival analysis web server based on gene expression profiles of diffuse large B‐cell lymphoma
Source: Cancer Med. 2020 Jan 9;9(5):1790–7. doi: 10.1002/cam4.2829 (PMC7050097; doi:10.1002/cam4.2829)
Supplement: Supplementary file 1 [file CAM4-9-1790-s001.docx]

**Supporting Information**

**Supplementary Figure**


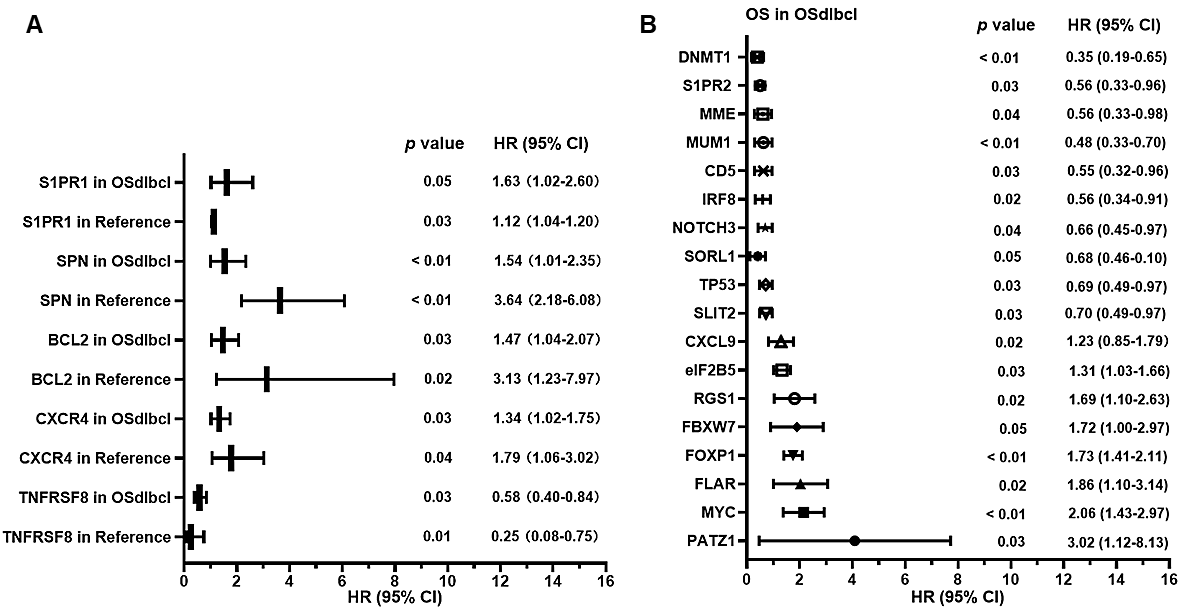


**Supplementary Figure 1. Forest plot evaluates the hazard ratio of gene expression between higher and lower for overall survival of DLBCL patients in OSdlbcl and in references.** (**A**) Five genes with HR in references and OSdlbcl. (**B**) The other 18 genes with HR in OSdlbcl.
